# Modern Perspectives on 30-Day Hospital Readmission Reviews

**DOI:** 10.7759/cureus.108135

**Published:** 2026-05-02

**Authors:** Rahul Kurapati, Asmaa Bousserhane, John Hon, Lucrezia Renzetti, Nai Chien Yeat, Nazish Ilyas, Vladimir Ornstein

**Affiliations:** 1 Internal Medicine, Lenox Hill Hospital, New York, USA; 2 Internal Medicine, Donald and Barbara Zucker School of Medicine at Northwell/Hofstra, New York, USA; 3 Internal Medicine, Northwell Health, New York, USA

**Keywords:** 30- day readmissions, health care cost, health care transitions, hospital medicine, preventable readmission

## Abstract

Background

Hospital readmissions are costly and impact healthcare quality. Accurate identification of preventable readmissions is crucial for targeted interventions.

Objective

We aimed to determine the proportion of preventable readmissions and identify the most common causes of readmission at a large academic medical center in New York City, United States of America.

Methods

We conducted a retrospective study of patients readmitted within 30 days of discharge from the hospital medicine service in 2022. Preventability was defined through a structured physician review process considering gaps in transitions of care, medication reconciliation, and clinical management.

Results

Of the 481 readmissions reviewed, 28% (133) were classified as preventable. Among the preventable readmissions, the most common causes were: medical management (26%, n=34), including clinical decisions and issues with medication reconciliation; misalignment of goals of care (20%, n=26), and lack of timely post-discharge follow-up (16%, n=21).

Conclusions

Approximately 28% of readmissions were preventable, highlighting opportunities for improvement in clinical decision-making, medication management, transition of care, and timely follow-up. Our findings support the use of validated readmission review methods and targeted interventions to address these key areas and reduce healthcare costs.

## Introduction

The rate of hospital readmissions is frequently used as a surrogate marker for quality of care. The "30-day readmission" rate is a key component of the scoring rubrics used by national quality reporting organizations such as the Centers for Medicare and Medicaid Services (CMS), U.S. News & World Report, Leapfrog, and Vizient. In addition to quality, the cost implications of readmissions weigh heavily on our healthcare system. According to the most recent Healthcare Cost and Utilization Project (HCUP) Statistical Brief from the Agency for Healthcare Research and Quality (AHRQ) [1], 3.6 million 30-day hospital readmissions (all-payer, all-cause) were documented in 2022, with an average readmission cost of $20,329. 

Prior efforts to reduce readmission costs have focused on high-risk patient populations. For instance, it is estimated that 16% of Medicare patients are readmitted within 30 days of discharge compared to the national average of 13.3% [1]. Sabbatini et al. [2] reported that 34% of Medicare patients are readmitted within 90 days. Policy interventions such as the Inpatient Quality Reporting Program and Medicare Hospital Readmissions Reduction Program have incentivized hospitals financially and reputationally to reduce the readmission rates of Medicare patients, with an additional focus on Medicare patients with high-risk diagnoses (e.g., Heart Failure, COPD, Pneumonia, Acute Myocardial Infarction, Coronary Artery Bypass, and Hip/Knee Arthroplasty). 

Van Walraven et al. [3] demonstrated that formulating a strategic approach to reducing 30-day readmissions requires two key steps. First, identifying the proportion of preventable readmissions. Second, understanding the common reasons for these readmissions, as the causes vary by hospital and by population. 

Prior studies [4,5] emphasize the need to understand the root causes of preventable readmissions before developing targeted interventions. Accurately determining the proportion of preventable readmissions has been challenging, with estimates ranging widely from 5% to 79% (median: 27%) [3]. While inherent subjectivity exists, adhering to validated methods, such as the structured physician review method described by Goldfield et al. [5], reduces variability and enhances reliability in identifying patterns of preventable readmissions [6]. Most validated tools focus on whether a readmission was preventable rather than identifying specific, modifiable institutional factors that contributed to it [7]. We believe a single-center retrospective cross-sectional study to (1) determine the proportion of preventable readmissions and (2) identify actionable causes amenable to institutional intervention can bridge this gap by combining the rigor of structured and validated physician review methods with institution-specific contextual knowledge, enabling identification of actionable causes. While our single-center design limits statistical generalizability, it maximizes practical generalizability: our transparent taxonomy and reproducible methodology can be adapted by other institutions to conduct similar assessments tailored to their contexts. 

## Materials and methods

Methods 

After obtaining institutional IRB exemption, we retrospectively reviewed 30-day readmissions occurring between January 1, 2022, and December 31, 2022. Eligible patients were initially discharged from the hospitalist service at Lenox Hill Hospital, a 480-bed academic medical center in New York City, and subsequently readmitted (inpatient or observation status) to any of the 21 hospitals within our nonprofit health system in New York State. The inclusion and exclusion criteria are listed in Table 1. 

**Table 1:** Inclusion and Exclusion Criteria

Inclusion Criteria	Exclusion Criteria
Age above 18 years	Discharged to an inpatient psychiatric facility on index admission.
Patients discharged home, sub-acute rehab, nursing home, acute rehab, long-term acute care hospital, or shelter	Discharged against medical advice (AMA) on index admission.
Patients readmitted within 30 days of discharge	Discharged to inpatient hospice on index admission.
Patients readmitted to any hospital within our hospital system	Readmitted for a scheduled elective procedure.
	Readmission for scheduled inpatient chemotherapy.

Data collection 

The hospital data analytics team used Tableau software (Tableau Software, Seattle, WA) to extract demographic and clinical data from the electronic medical records (Veradigm Inc., Chicago, IL). Variables abstracted included age, gender, LACE score (Length of stay, Acuity of admission, Comorbidity, and Emergency department use) [8], date of index admission, length of stay, date of readmission, insurance status, attending of service, discharging floor/unit, as well as information about transitions of care (e.g., date and time of follow-up appointments if present at the time of discharge). Please see Appendix A for a full list of variables. 

Outcome measures 

The primary outcome measure was the proportion of 30-day readmissions deemed to be preventable. Because definitions of "preventability" vary, we used a practical definition of "potentially preventable readmissions" derived from Goldfield et al. [5]

We classified readmissions as preventable if one of the following could reasonably have prevented the readmission: (1) a different medical plan, (2) a better transition of care plan, or (3) improved communication with the emergency department on return. 

Our secondary outcome was to identify specific causes for both preventable and unpreventable readmissions. 

Process for case review of preventability and identification of underlying causes

Our team included five hospitalists and a project manager. Our project manager downloaded data from a Tableau dashboard monthly and stored the data in a HIPAA-compliant institutional OneDrive folder (Microsoft Corp., Redmond, WA) to facilitate asynchronous collaboration. An average of 50 readmissions each month met the inclusion criteria. These cases were divided equally among the five hospitalists for review. None of the team members reviewed readmissions for patients they were attending of record during the index admission. 

The reviewers had access to key data points abstracted from the medical record (Appendix A) and to the patient’s medical record through our electronic medical records. The reviewers were tasked with a) determining if readmission was preventable and (b) determining the cause for readmission. To standardize case review, we developed a taxonomy of 15 readmission attributions with explicit criteria for classification (Table 2). Each attribution was designated as preventable or non-preventable based on whether alternative management, improved transitions of care, or enhanced communication could reasonably have prevented readmission. This pre-specified list was created using case review data from the 12 months preceding this study (i.e., readmissions from 2021). 

**Table 2:** Definitions used by the reviewers when determining the cause of readmission and determining preventability

Attribution​	Preventable (Yes/No)	Criteria ​
Medical management (decision making)​	Yes​	Issues with clinical judgment, treatment choices, and failure to adhere to guidelines (excluding medication reconciliation, medication access-related cases).
Medication reconciliation and access	Yes​	Encompasses issues with the accuracy of medication reconciliation, failure to identify barriers to access, and failure to send correct prescriptions.​
Lack of timely follow-up appointment​	Yes​	Follow-up appointment arranged with a date and time within 2 weeks of discharge (primary care physician or subspecialist).
Did not need index admission​	Yes​	Index admission did not meet inpatient criteria. Should have been admitted under observation status/treat and Release from the emergency room on index admission.
Hospice​	Yes​	Could the patient have been directly admitted to an inpatient hospice unit?
Discussion in the emergency department	Yes​	Lack of discussion between the discharging physician and the emergency room provider before readmission. Reasonable expectation that information/context provided by the discharging physician regarding the index admission would have prevented re-admission.
Dialysis​	Yes​	Readmission for non-urgent dialysis.
Communication​	Yes​	Reasonable expectation that better communication with the patient/caregiver/sub-acute rehab at the time of discharge would have prevented readmission​.
Progression of disease/malignancy​	No​	Examples: Admission for new cancer symptoms (new effusion, new ascites, new bone pain), readmission for urgent dialysis, new cirrhotic symptoms.
New disease/different process​	No​	Returning for a reason unrelated to the index admission.
Patient adherence/preference​	Yes/No​	Patient’s failure to adhere to recommended therapy/intervention/disposition (e.g., refusing outpatient parenteral antibiotics, refusing sub-acute rehab).​
Alcohol​	No​	Readmission due to alcohol intoxication/withdrawal despite the provision of adequate substance use referral.
Recurrent disease​	No​	Recurrence of known problem (e.g., recurrent hepatic encephalopathy despite optimized meds).​
Social determinant​	No​	Examples: No safe disposition option from the emergency room; unable to travel to a follow-up appointment; poor wound care or inability to store insulin because of an undomiciled​ status.
Adverse effects of therapy​	No​	Adverse events related to medical therapy (e.g., side effects of medication)​.
Palliative​ care consultation	Yes/No​	Goals of care were not fully addressed on index admission.
Consultant recommendations ​	Yes/No​	Examples: inpatient vs outpatient endoscopy; inpatient vs outpatient long-term pleural catheter placement.
Transfusion/infusion​	Yes/No​	Example: readmission for blood product transfusion in the setting of known chronic hematologic disease. Avoidable if the patient is hemodynamically stable on readmission. Unavoidable if hemodynamically unstable or has severe symptoms that cannot be managed on an outpatient basis.

The team discussed cases adjudicated as preventable or equivocal during the weekly meeting before final assignment of preventability and cause of readmission. If there was disagreement among the reviewers, it was resolved with a simple majority. Clearly unpreventable cases were marked as such after a first-pass review. 

## Results

**Figure 1:**
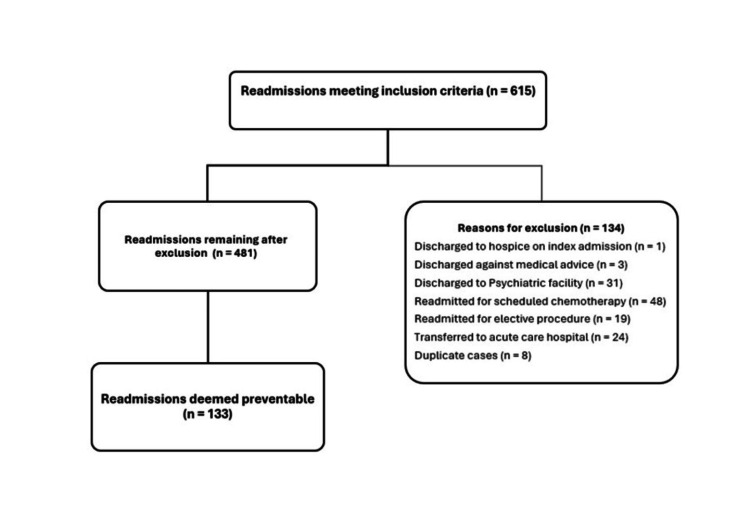
Flow Diagram Depicting Selection Process

Of the 615 readmissions meeting the inclusion criteria, 134 were excluded based on the exclusion criteria (Figure 1). The most common exclusions were elective chemotherapy admissions (n = 48), elective procedures (n = 19), psychiatric facility discharges (n = 31), and transfers to acute care (n = 24). The readmission committee reviewed 481 cases, of which 133 (28.06%) were deemed preventable.

**Figure 2:**
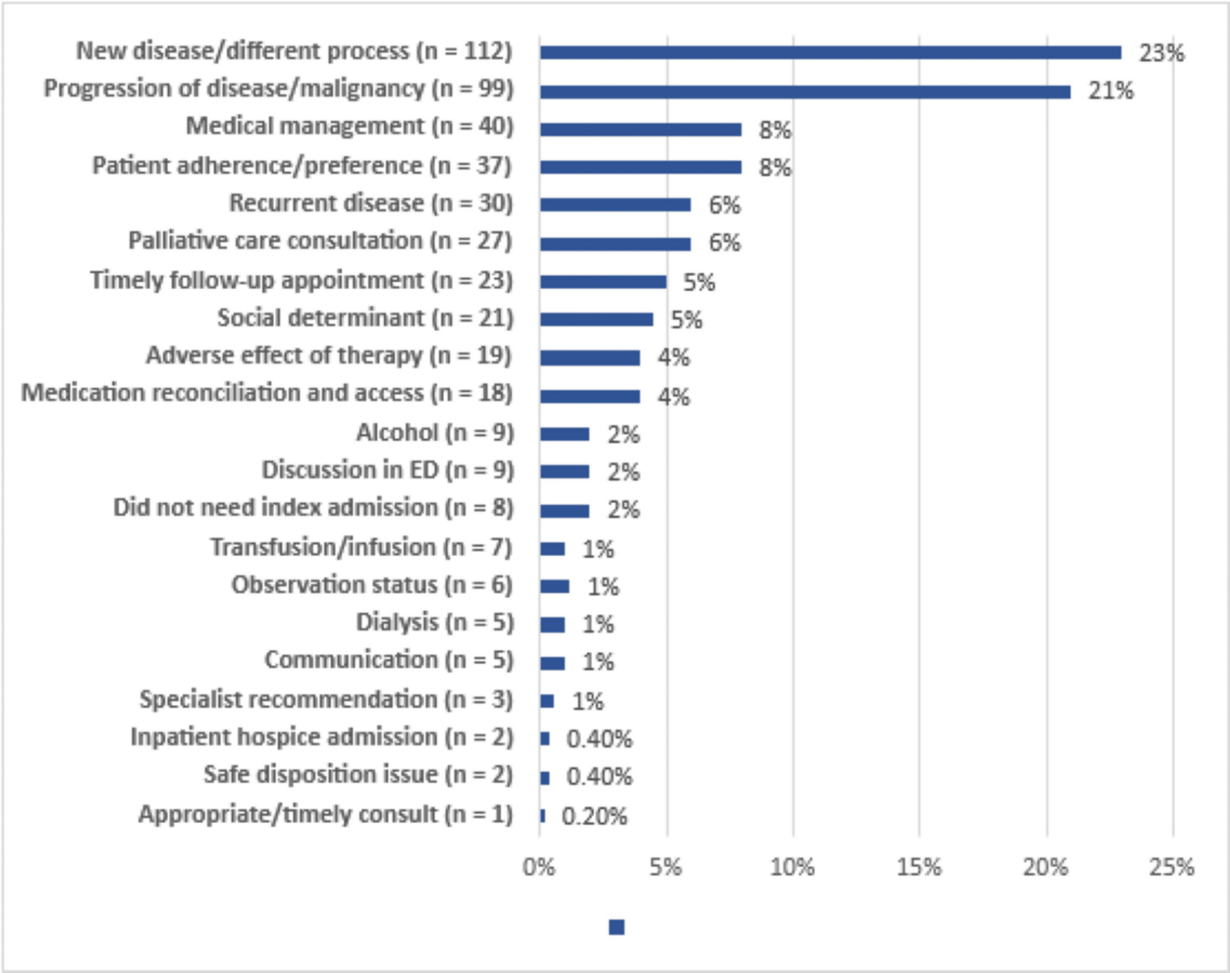
Causes of Readmissions Illustrates the most common causes of readmissions (both preventable and non-preventable).

The most common cause of readmission was a new disease or a process unrelated to the index admission (n = 112, 23%) (Figure 2). Other notable causes for readmission include progression of disease (n = 99, 21%), medical management (n = 40, 8%), patient adherence/preference (n = 37, 8%), recurrent disease (n = 30, 6%), and "palliative care" - defined as failure to adequately address goals of care on the index admission (n = 27, 6%). 

**Figure 3:**
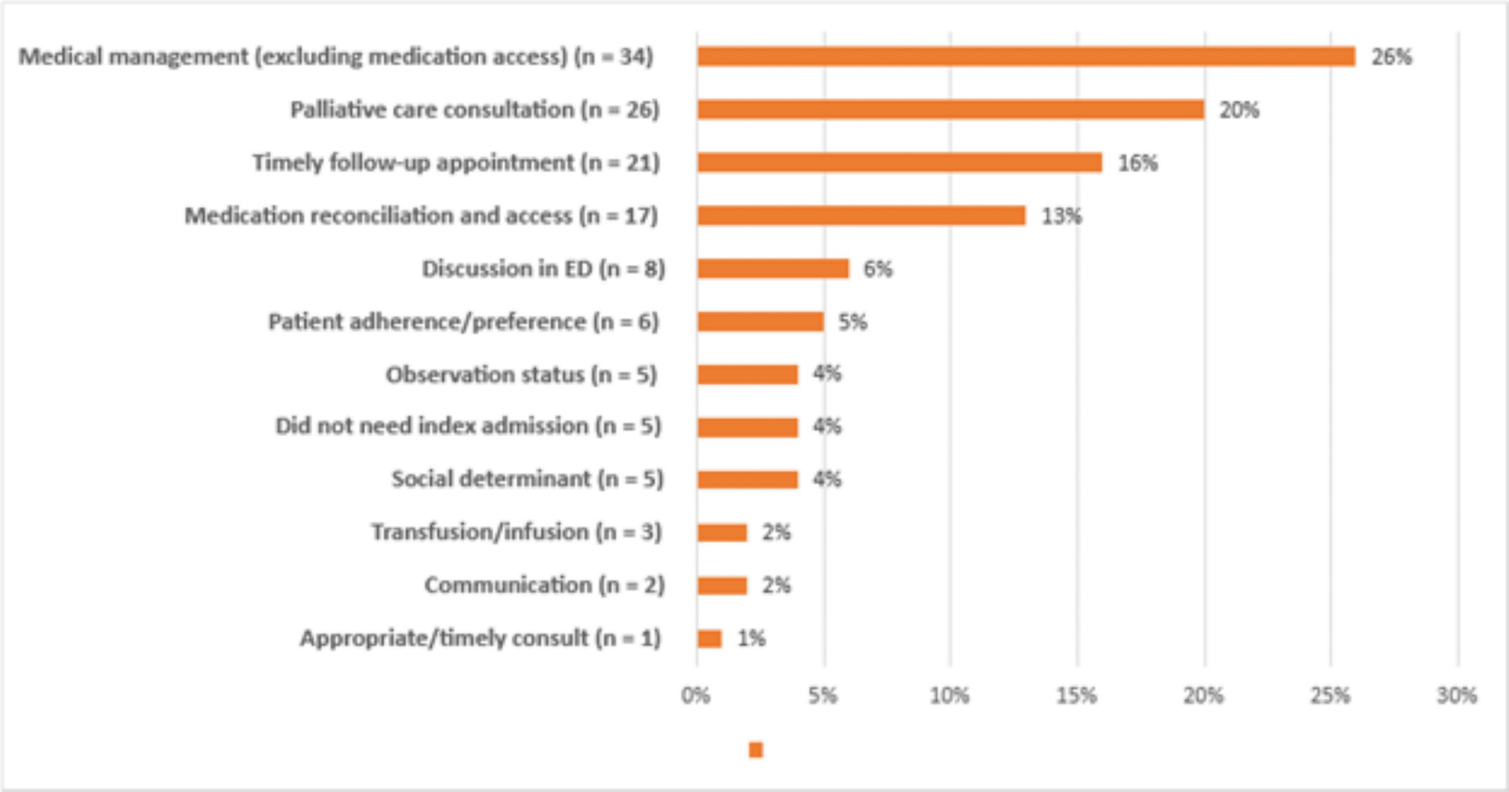
Preventable Readmissions Illustrates the most common causes of preventable readmissions.

Among the 133 readmissions deemed preventable, the most common cause was medical management (excluding medication reconciliation and access-related) (n = 34, 26%), followed by lack of palliative team involvement (n = 26, 20%), lack of timely outpatient follow-up (n = 21, 16%), and medical reconciliation/medication access (n = 17, 13%). Cumulatively, medical management (both medication-related and non-medication-related) accounted for n = 51, 39% of preventable readmissions (Figure 3).

## Discussion

We found that 28% of readmissions were preventable with medical management, palliative care consultation, and a lack of timely post-discharge follow-up appointment as the primary causes. The majority (72%) were deemed non-preventable, primarily due to disease progression (23%) or new unrelated conditions (21%). Our study's 28% preventability falls within the estimates of preventable hospital readmissions, which have ranged between 5% and 79% using various inclusion criteria [3]. Most studies suggest the rate of preventable readmissions clusters around 20 to 75% of all hospital readmissions [3,9,10]. 

Medical management-related reasons accounted for 26% of all preventable readmissions, and 13% were related to medication reconciliation and medication access. Van der Does et al. [11] reported that medical management and medication reconciliation/access each accounted for 27% of preventable readmissions, similar to our findings. This distinction is important because readmissions attributable to issues with clinical diagnosis and decision-making can be decreased by employing strategies that target physician competency, whereas those attributable to system-based practice, such as medication access issues, may be more likely to respond to system-wide quality improvement initiatives. Previous literature is mixed regarding the effectiveness of medication reconciliation interventions. A randomized controlled trial performed in 2021 found that medication reconciliation alone did not decrease readmissions in older patients at risk for polypharmacy [12]. However, a 2021 meta-analysis found that medication review in combination with medication reconciliation, patient education, professional education, and transitional care was associated with decreased rates of readmissions [13]. This suggests that a multi-modal approach, inclusive of attention to medication reconciliation and access during transitions of care, should be the standard of care for improving individual patient safety. 

Misalignment of goals of care accounted for 19% of all preventable readmissions. This occurred when goals of care were not fully addressed during the index admission, leading to a future readmission. Early palliative care involvement for patients at the end of life is an actionable and necessary intervention during hospitalization. Furthermore, appropriate involvement of palliative care for these patients has been shown in multiple studies to prevent readmissions, including for those for whom hospice referral may be appropriate [14,15].

Access to timely post-discharge follow-up accounted for 16 percent of all preventable readmissions. This refers to the lack of a timely follow-up appointment with a primary care provider or specialist within 14 days of discharge, scheduled before discharge, as defined by CMS. A 2022 study showed that a primary care physician visit within 7 or 14 days of hospital discharge was associated with lower rates of 30-day hospital readmissions [16]. This is also reflected in the importance given by CMS in adding billing codes for transition of care services for follow-up visits, 7 to 14 days after hospital discharge. However, gaps remain in adherence to post-discharge visits. Possible explanations include geographic accessibility, patient education, challenges with securing timely appointments, and insurance status. 

Several limitations merit consideration when interpreting its results. The study was designed as a retrospective study, which may result in unmeasured biases. Although we used a consensus-based approach for equivocal cases, we did not formally assess inter-rater reliability. Additionally, the study focused on patients discharged from a single institution, which limits generalizability; however, we believe this limitation was partially mitigated by reviewing readmissions to the 21 hospitals within our healthcare system [17]. 

Furthermore, the evaluation of preventable readmissions was conducted by a select group of hospitalist reviewers involved in the hospital's readmission reduction committee; their unique expertise in hospital medicine may not extend to professionals in other specialties, thus affecting the study's applicability beyond this field. 

Despite these limitations, the importance of institutional review of readmissions using validated techniques remains a cornerstone of academic hospital medicine. As the literature on transitions of care matures and as payer models are increasingly geared towards home-based and preventative care, up-to-date analyses of readmission data can help guide interventions and improve patient outcomes. 

## Conclusions

In this retrospective review of 481 30‑day readmissions, we found that 133 readmissions were preventable. Medical management and medication reconciliation/access accounted for the largest share of preventable cases; goals of care misalignment and lack of timely follow‑up appointments were also leading contributors. These results underscore that a meaningful portion of readmissions reflects remediable gaps in care rather than inevitable disease progression. Addressing these gaps requires both clinician-focused interventions--improving diagnostic and treatment decision-making--and system-level changes, including robust medication access processes, routine palliative care engagement when appropriate, and guaranteed follow-up within 7-14 days. While our single-center design limits generalizability, our findings align with broader literature and identify concrete, actionable targets for institutional quality improvement.

Our study's primary contribution is twofold: we apply a pragmatic, multidisciplinary, and validated case‑review process to quantify preventable readmissions at an institutional level, and we identify specific causes that quality improvement efforts can target. By categorizing readmissions using a reproducible taxonomy, our work translates readmission review from an abstract quality metric into concrete intervention priorities. Future work should prospectively test bundled interventions informed by these findings, combining medication reconciliation with access solutions, palliative consult triggers, and mechanisms to secure timely outpatient appointments. Multisite evaluations and ongoing institutional review with clinician feedback will be essential to confirm effectiveness, scale successful strategies, and sustain reductions in preventable readmissions.

## Appendices

Appendix A

Variables abstracted from the electronic medical record (Table 3)

**Table 3:** Variables Abstracted from Electronic Medical Record

Category	Variable
Medical record number	-
Index admission	Account no.
Index admission	Date of admission
Index admission	Date of discharge
Index admission	Length of stay
Index admission discharging floor/unit	-
Index admission discharge disposition (e.g. routine discharge, SAR, SNF, psychiatric facility)	-
Index admission	Attending of record
Index admission	Discharge diagnosis
Care provider (primary care physician) follow-up	-
Date and time of primary care physician appt (if present)	-
Date and time of specialty appt 1 (if present)	-
Date and time of specialty appt 2 (if present)	-
Readmission account no.	-
Readmission	Date of admission
Readmission	Date of discharge
Readmission	Account no.
Index admission	LACE score
Primary care physician (if identified on index admission)	-
Index admission	Palliative consultation (yes/no)
Index admission	GOC conversation documented (yes/no)
Age in years	-
Insurance	-
